# Enhancing Radiomics Reproducibility: Deep Learning-Based Harmonization of Abdominal Computed Tomography (CT) Images

**DOI:** 10.3390/bioengineering11121212

**Published:** 2024-11-30

**Authors:** Seul Bi Lee, Youngtaek Hong, Yeon Jin Cho, Dawun Jeong, Jina Lee, Jae Won Choi, Jae Yeon Hwang, Seunghyun Lee, Young Hun Choi, Jung-Eun Cheon

**Affiliations:** 1 Department of Radiology, Seoul National University Hospital, 101 Daehak-ro, Jongno-gu, Seoul 03080, Republic of Korea; rusia928@naver.com (S.B.L.); djc0105@gmail.com (J.W.C.); jyhwang79@gmail.com (J.Y.H.); seunghyun.lee.22@gmail.com (S.L.); choiyounghun@gmail.com (Y.H.C.); cheonje@snu.ac.kr (J.-E.C.); 2 CONNECT-AI R&D Center, Yonsei University College of Medicine, Seoul 03080, Republic of Korea; hyt0205@gmail.com (Y.H.); jdwekdns1@gmail.com (D.J.); qqwwdj@yonsei.ac.kr (J.L.); 3 Department of Radiology, Seoul National University College of Medicine, 103 Daehak-ro, Jongno-gu, Seoul 03080, Republic of Korea; 4 Brain Korea 21 PLUS Project for Medical Science, Yonsei University, Seoul 03080, Republic of Korea; 5 Institute of Radiation Medicine, Seoul National University Medical Research Center, 103 Daehak-ro, Jongno-gu, Seoul 03080, Republic of Korea

**Keywords:** radiomics, CT acquisition, artificial intelligence, reproducibility, quality control

## Abstract

We assessed the feasibility of using deep learning-based image harmonization to improve the reproducibility of radiomics features in abdominal CT scans. In CT imaging, harmonization adjusts images from different institutions to ensure consistency despite variations in scanners and acquisition protocols. This process is essential because such differences can lead to variability in radiomics features, affecting reproducibility and accuracy. Harmonizing images minimizes these inconsistencies, supporting more reliable and clinically applicable results across diverse settings. A pre-trained harmonization algorithm was applied to 63 dual-energy abdominal CT images, which were reconstructed into four different types, and 10 regions of interest (ROIs) were analyzed. From the original 455 radiomics features per ROI, 387 were used after excluding redundant features. Reproducibility was measured using the intraclass correlation coefficient (ICC), with a threshold of ICC ≥ 0.85 indicating acceptable reproducibility. The region-based analysis revealed significant improvements in reproducibility post-harmonization, especially in vessel features, which increased from 14% to 69%. Other regions, including the spleen, kidney, muscle, and liver parenchyma, also saw notable improvements, although air reproducibility slightly decreased from 95% to 94%, impacting only a few features. In patient-based analysis, reproducible features increased from 18% to 65%, with an average of 179 additional reproducible features per patient after harmonization. These results demonstrate that deep learning-based harmonization can significantly enhance the reproducibility of radiomics features in abdominal CT, offering promising potential for advancing radiomics development and its clinical applications.

## 1. Introduction

Radiomics provides a quantitative approach to image analysis. By extracting numerous quantitative features from medical images and studying their correlations with clinical or genetic features, radiomics holds promise for diagnosis, stratification, and prognostic prediction [1,2,3]. However, the low reproducibility of radiomics features is a significant obstacle to the application of radiomics in real-world clinical settings. Variability in image acquisition, preprocessing, and feature extraction can introduce substantial variability in radiomics features, leading to unreliable results and reduced clinical utility [4].

The reproducibility of radiomics is affected by each step of the radiomics model building process, including image acquisition, preprocessing, and feature extraction. Minor variations in image acquisition parameters, such as slice thickness and reconstruction kernel, can significantly affect the extracted features [5]. Additionally, differences in preprocessing steps further contribute to variability [6,7]. As radiomics requires a substantial number of images to construct models, obtaining a large number of homogeneous images from a single institution is often impractical. Therefore, inhomogeneous images from different machines, vendors, and institutions are commonly used for model development. However, this introduces various confounding factors that affect the stability of radiomic features, including vendor and machine types, acquisition parameters, and reconstruction methods [4,8,9,10,11,12]. To address this issue, the standardization of medical images is essential for stabilizing radiomics features.

To overcome the challenge of low reproducibility, efforts have been made to standardize radiomics protocols across different institutions and imaging systems. A promising approach is the use of artificial intelligence (AI) to standardize images through deep learning-based image transformation. AI potentially identifies and corrects sources of variability, leading to more accurate and reliable radiomics analysis. One of the most notable deep learning algorithms in medical imaging is the generative adversarial networks (GANs). GANs can generate synthetic images or convert low-resolution images into high-resolution images. A previous report introduced a deep learning-based image conversion model that significantly improved the reproducibility of radiomics features in CT scans using body phantoms [13]. The developed algorithm demonstrated an 83.3% improvement in the concordance correlation coefficient (CCC) for synthetic images compared with the original images. However, to deploy image conversion algorithms in clinical practice, it is essential to develop and validate an algorithm using real human data.

In this study, we aimed to develop and evaluate the effectiveness of a deep learning-based CT image harmonization algorithm for improving the reproducibility of radiomics features in abdominal CT. Specifically, we sought to compare multiple reconstruction protocols derived from the same CT scan and to harmonize images from these varying protocols into a single target protocol using our deep learning model.

## 2. Materials and Methods

This retrospective study was approved by the Institutional Review Board of Seoul National University Hospital, and the need for informed consent was waived (IRB No. 2108-139-1246).

### 2.1. Study Design

This study retrospectively evaluated a deep learning-based harmonization algorithm for enhancing the reproducibility of radiomics features in abdominal CT scans. Data were collected from patients who underwent dual-energy CT (DECT) scans, with 117 patients (142 scans) used for training and internal validation and 63 scans from an independent cohort for external validation. Multiple reconstruction protocols, including filtered back projection (FBP), iterative reconstruction (IR), and virtual monoenergetic imaging at various energy levels, were used. A hierarchical feature synthesis-based generator network with pixel unshuffling and sequential spatial-channel attention was employed, paired with a U-Net-style discriminator for CT image harmonization. Radiomics analysis focused on 455 features extracted from ten manually segmented regions of interest (ROIs) across organs. Reproducibility was assessed via intraclass correlation coefficients (ICCs) using region- and patient-based analyses, with features showing high redundancy excluded from the final analysis.

### 2.2. Study Population

We retrospectively collected data from patients who underwent abdominal CT. Only CT images acquired using dual-energy (DE) scans were included in the training and internal validation sets. We excluded CT images with severely degraded image quality. Finally, for the training and internal validation sets, we collected data from 117 patients who underwent 142 contrast-enhanced abdominal CT examinations at a single tertiary hospital between March 2021 and July 2021 (Table 1). The training and validation sets used in this study are the same as those used in a previous study [14]. For the external validation set, we separately collected 63 contrast-enhanced abdominal CT examinations (in 63 patients) using different CT machines at the same tertiary center between August 2021 and May 2022 (Table 1).

### 2.3. Image Acquisition and Reconstruction

The training and internal validation sets were scanned using Somatom Force (Siemens Medical Systems, Erlangen, Germany) with a DE scan. The DECT images of each patient were reconstructed as follows: filtered back projection (FBP), iterative reconstruction (IR) with a strength of 3, virtual monoenergetic images with 40 keV (M40), 60 keV, 80 keV, and optimum contrast. CT images were acquired using Somatom Definition Flash (Siemens Medical Systems, Erlangen, Germany). The DECT images in the external validation set were reconstructed using FBP, IR, M40 and 70 keV. The detailed CT parameters are summarized in Table 2.

### 2.4. Deep Learning Architecture

Deep learning architectures for the GANs were developed based on the findings of previous studies [13,14]. A previous study introduced a Hierarchical Feature Synthesis (HFS) module-based generator network architecture to improve the reproducibility of radiomics features in abdominal phantom CT images [13]. We advanced the HFS module-based generator network by substituting two-dimensional spatial average pooling with pixel unshuffling to preserve spatial information [14,15]. Pixel unshuffle enables models to handle images with lower resolution while maintaining essential spatial information, enhancing performance in detailed image reconstruction tasks. Furthermore, we used a U-Net-style discriminator to provide a more sophisticated image translation for CT standardization [16]. The U-Net architecture discriminator network provides pixel-wise feedback. Thus, it is remarkable at detecting spatial and textural anomalies. Additionally, we demonstrated that the sequential application of spatial and channel attention layers improved image translation performance more than the parallel application of spatial and channel attention layers [17]. We retained the generator architecture by applying sequential spatial and channel attention to the HFS module, and the U-Net-style discriminator structure was used. In this study, the input data consist of CT images reconstructed using various methods, with the ground truth image for harmonization being an IR image with strength level 3. Detailed information of the deep learning architecture and experimental methods is provided in the Supplementary Materials (Figures S1 and S2).

### 2.5. Radiomics Analysis

**Figure 1:**
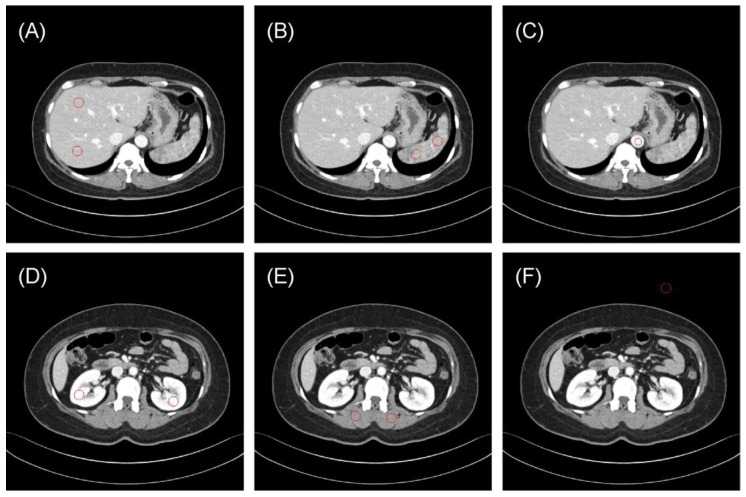
Representative examples of regions of interest (ROIs, red circle) for evaluating the reproducibility of radiomics features. (**A**) Liver parenchyma, (**B**) spleen, (**C**) vessels (aorta), (**D**) bilateral kidneys, (**E**) paraspinal muscles, and (**F**) air. The typical ROI sizes are 120 mm^2^ for vessels and 240 mm^2^ for other regions, with adjustments to 50 mm^2^ and 100 mm^2^ in some instances depending on body size.

One radiologist (S.B.L., 8 years of experience in image interpretation) drew 10 regions of interest (ROIs) to evaluate the reproducibility of radiomics features (Figure 1): the liver parenchyma (two ROIs in the portal phase), spleen (two ROIs), bilateral kidneys (two ROIs), paraspinal muscles (two ROIs), vessels (one ROI) and air regions (one ROI). The Pyradiomics library version 2.2.0 was used to extract radiomics features from each ROI of the original and synthetic images [18].

The core radiomics feature set comprised 91 features with six different matrices: (1) 18 first-order statistical features, (2) 22 texture features derived from the co-occurrence matrix, (3) 16 features based on the gray-level run length matrix (glrlm), (4) 16 features based on the gray-level size zone matrix (glszm), (5) 14 features based on the gray-level dependence matrix, and (6) 5 features based on the neighboring gray-tone difference matrix. The core radiomics features were extracted based on Hounsfield unit intensity images, and additional radiomics features were derived by further processing the images with four different wavelet filters. Therefore, we extracted 455 radiomics features consisting of 30 feature groups.

### 2.6. Redundancy Feature Exclusion

Several radiomics features are redundant [8]. Therefore, we identified and excluded redundant features from our analyses. We defined a redundant feature group if more than half of the features in a class exhibited an intraclass correlation coefficient (ICC) ≥ 0.85 across all protocol types in the training dataset. The high ICC across all protocols implies that these features fail to capture differences between protocols. These groups were subsequently excluded from analysis if they were present in over half of the patients within our external validation set. Consequently, the following four feature groups were excluded: “original_firstorder”, “wavelet-low-low (LL)_firstorder”, “wavelet-LL_glrlm”, and “wavelet-LL_glszm”. In total, 387 features were retained for analysis.

### 2.7. Statistical Analyses

In our study, “reproducibility” refers to the consistency of radiomic features measured across different imaging protocols, with protocols serving as the raters. We aimed to examine this reproducibility both across patients (per-ROI) and within each patient (per-Patient) to capture variability due to protocol differences, rather than observer variability or repeated measurements by the same observer.

ICCs were used to evaluate the reproducibility of radiomics features. The reproducibility was evaluated using two distinct methodologies: region- and patient-based. Radiomics features with an ICC of ≥0.85 were considered reproducible, and their percentage was calculated [9,19]. In the region-based method, reproducibility was assessed for each organ. When two ROIs from the same organ were analyzed, the number of features exhibiting an ICC ≥ 0.85 in both ROIs was recorded. In the patient-based method, the ICC was computed independently, without considering ROIs from the same organ.

All statistical analyses were performed using the Python package (pingouin 0.5.2; Python Software Foundation). A *p* value < 0.05 was considered statistically significant. To visually demonstrate the effect of harmonization, we constructed a standard deviation map.

## 3. Results

### 3.1. Region-Based Reproducibility Analysis

The reproducibility of the radiomics features in each ROI before and after image harmonization is summarized in Table 3. In internal validation, image harmonization significantly increased reproducibility for radiomics features, with an ICC ≥ 0.85 across all ROIs, except for air. Notably, the vessel features exhibited the most substantial increase in reproducibility, increasing by 41% from 36% before harmonization to 77% thereafter. Although the liver parenchyma demonstrated the lowest increase among the tissues examined, it still showed significant improvement, with reproducibility increasing from 24% before harmonization to 57% after a 33% increase. Additionally, enhanced reproducibility was observed in the spleen (21% to 60%), kidney (22% to 63%), and muscle (42% to 68%). By contrast, the reproducibility in air slightly decreased, from 94% to 89%.

External validation yielded similar results, with the highest reproducibility observed for the vessel features, improving from 14% to 69%. Increases were also noted in the spleen (18% to 62%), kidney (15% to 63%), and muscle (24% to 55%). The liver parenchyma also showed enhanced reproducibility, increasing from 19% to 51%. Although the reproducibility for air slightly decreased from 95% to 94%, the actual decrease in the number of features was minimal, from 369 to 365, indicating a negligible difference, and the reproducibility was higher than that in the internal validation.

### 3.2. Patient-Based Reproducibility Analysis

**Figure 2:**
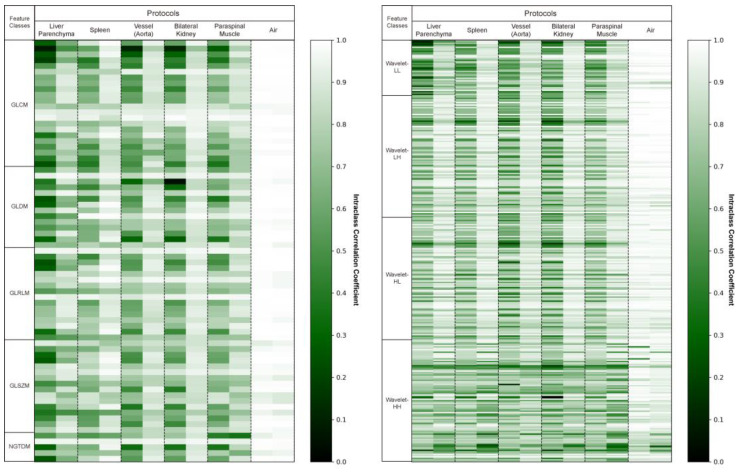
Intraclass correlation coefficient (ICC) heatmaps for all radiomics features in the original and synthetic images. The heatmaps display the ICC values of the 387 radiomics features averaged across two identical regions of interest. This visual representation indicates how the ICC values change before and after harmonization. Higher ICC values are depicted as brighter.

For the external validation cohort, we drew the same 10 ROIs (two liver parenchyma, two spleen, two vessels, two kidneys, two muscles, and two air regions) for all 63 patients and extracted 387 radiomics features from each. We evaluated the reproducibility of these radiomics features before and after applying deep learning-based harmonization in the external validation cohort. An average of 71 ± 19 (18%) radiomics features were reproducible (ICC ≥ 0.85) before harmonization. After harmonization, an average of 250 ± 61 (65%) radiomics features was found to be reproducible. We experimentally found that harmonization resulted in an average of 179 additional radiomics features per patient that were reproducible. Figure 2 shows the ICC values for each patient before and after image harmonization as a heatmap.

### 3.3. Standard Deviation Map

**Figure 3:**
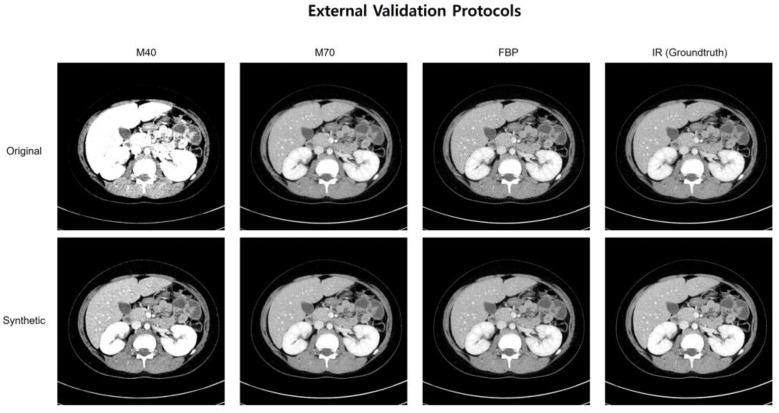
A representative case of abdominal CT before and after harmonization in the external validation set.

**Figure 4:**
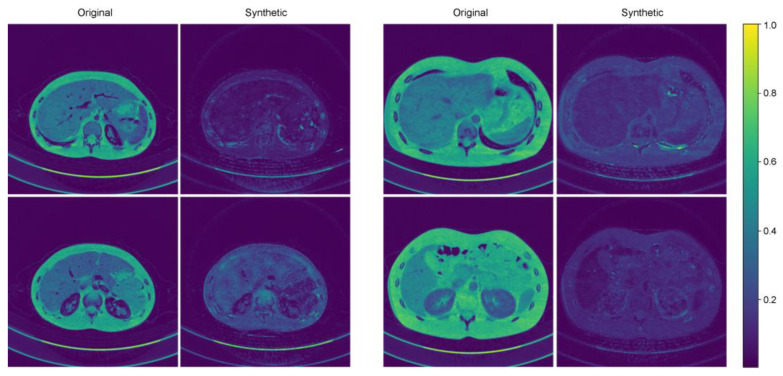
Two representative examples of standard deviation maps.

Figure 3 shows representative cases of abdominal CT before and after harmonization in the external validation set. Each image is displayed with a window width of 300 and a level of 40 to ensure consistent visualization across the comparison. In the external validation, all the voxels in the four protocols (M40, M70, FBP, IR) had the same pixel-wise correspondences. Therefore, by calculating the pixel-wise differences across the four protocols and visualizing their standard deviations, we can clearly illustrate the harmonization effect. Six combinations were used to calculate the pixel-wise differences among the four protocols, and the standard deviations of these six differences were used to visualize the effect. The results of the standard deviation map are shown in Figure 4.

## 4. Discussion

The results of our study demonstrate that the developed deep learning model can improve the reproducibility of radiomics features in real human CT images. Using a deep learning-based image conversion approach, we successfully reduced the variability in radiomics features resulting from differences in CT machines and post-processing methods. The results of this study indicate an enhancement in the reproducibility of radiomics features extracted from CT images. This improvement raises the prospect of a more extensive application of radiomics in practical clinical settings.

Most radiomics models published to date are limited by the lack of large, standardized datasets and the absence of clinical validation [20]. Ideally, radiomics features should be independent of image acquisition parameters or protocols [20]. However, several previous studies have pointed out the poor reproducibility of CT radiomics features across different CT acquisition parameters, reconstruction methods, and CT scanners [4,8,10,21].

In our previous study, we developed an image conversion algorithm using a residual feature aggregation network to harmonize images from varying CT protocols and evaluated the algorithm to improve the reproducibility of radiomics features extracted from CT scans across different protocols, reconstruction techniques, and scanners [13]. The algorithm was developed, and its performance was validated using an abdominal phantom in the previous study [13]. However, to facilitate its application in real clinical settings, it was necessary to adjust the algorithm using actual patient abdominal images and to test its performance accordingly. In the prior phantom-based study, the deep learning model demonstrated a substantial improvement in reproducibility, achieving an 83.3% increase in the concordance correlation coefficient (CCC) for synthetic images compared to the original [13]. In this current study, using real patient abdominal CT images, we observed an increase in the proportion of features with an ICC value ≥ 0.85 from 18% to 65%. This finding underscores the potential of the previously developed algorithm for application in clinical settings.

Several studies on enhancing the reproducibility of radiomics using deep learning, particularly GANs, have compared radiomics features with a CCC ≥ 0.85 before and after applying deep learning to demonstrate reproducibility improvement [22,23,24]. However, Robert et al. revealed that intra-CT analysis showed a wide range of reproducibility, depending on parameter adjustments [8]. This indicates that several radiomics features exhibit redundant properties. Therefore, it is crucial to focus on improving the reproducibility of essential features, excluding redundant features, rather than enhancing their reproducibility across all radiomics features. Unlike other studies and our previous phantom study, this study excluded redundant features and assessed reproducibility improvement solely for the necessary radiomics features [13].

In this study, we used ICCs to evaluate the reproducibility of radiomics features. However, there are concerns about the use of ICC to assess reproducibility and agreement of radiomics features [25]. When utilizing ICC as a reliability metric, researchers should pay close attention to selecting the most appropriate ICC form, as an inappropriate choice may yield numerically similar ICC values but lead to significantly different or misleading interpretations. Additionally, none of the reviewed studies conducted sample size estimation for ICC calculations, which, while not mandatory, could improve the precision of ICC measurements. Furthermore, confidence intervals for ICC values should be reported, as they are essential for assessing the precision and reliability of the estimates. These intervals are necessary to estimate the precision of the reliability estimates.

Efforts to enhance the reproducibility of CT radiomics can be divided into image and feature domain strategies. [20]. The harmonization strategy in the image domain includes the development of standardization guidelines and the utilization of raw image datasets [20]. By creating guidelines that regulate the type of scanner, protocol, imaging parameters, and reconstruction method, standardized image acquisition can be achieved. For example, the Food and Drug Administration released imaging guidelines to optimize the quality of imaging data for clinical trials supporting the approval of drugs and biological products [26]. The EARL (the European Association of Nuclear Medicine Research Ltd., Vienna, Austria) program, initiated by the EANM (the European Association of Nuclear Medicine, Vienna, Austria), encompasses workflow, including scan acquisition, image processing, and image interpretation [27]. Pfaehler et al. explored the effects of harmonizing image reconstructions on the reproducibility of radiomics features, revealing that adopting EARL-compliant image reconstruction successfully standardized the radiomics features [28]. However, while such standardized protocols may work for specific projects, they lack flexibility for broader applications in heterogeneous, retrospective datasets commonly used in radiomics studies. Additionally, evolving hardware and software for image acquisition limit the practicality of strictly standardized guidelines. Another approach is to use raw sensor-level image data, such as sonograms in CT or k-space data in MRI, which may reduce radiomics variability introduced during image reconstruction [29]. However, this approach is challenging for retrospective studies and does not fully address variability from acquisition parameters. Feature-domain harmonization strategies, including reproducible feature selection and normalization techniques, aim to stabilize radiomics features [6,30,31,32,33,34]. Collaborative efforts like the Image Biomarker Standardization Initiative (IBSI) have established protocols to standardize radiomics computations, although these methods cannot fully account for variations caused by differences in scanners, protocols, or reconstruction methods [34]. Furthermore, robust radiomics features may vary across diseases or organs, limiting their universal application in research and clinical settings.

In this study, we developed a deep learning model for harmonizing CT images to improve the reproducibility of CT radiomics features. Image harmonization using a deep learning model has several advantages. As multicenter retrospective studies are increasingly being conducted, there is a growing need for post hoc harmonization rather than harmonization in the early steps of image acquisition and analysis. AI-based techniques are promising post hoc image harmonization techniques aimed at mitigating the influences arising from the utilization of center-specific devices and imaging protocols. The Comprehensive Analysis of Bioinformatics Toolbox (ComBat) is a statistical method developed to correct for batch effects. ComBat is specifically designed to reduce batch effects caused by various factors. It is widely used in harmonizing medical imaging; however, there are several limitations to deep learning-based image conversion. Owing to the inherent characteristics of statistical techniques, the ComBat method requires information regarding the sources of variation [35]. Therefore, it may not exhibit adequate performance on unknown datasets. By contrast, deep learning-based image conversion can adapt to diverse datasets, although it requires substantial amounts of training images.

Our study has some limitations. First, our external validation was conducted using a single type of CT scanner and image reconstruction methods. Therefore, our findings may not be generalizable to other types of CT machines or imaging protocols. Further studies are required to expand the external validation of our image conversion model to include multiple types of CT machines and various imaging protocols. Second, we did not assess the reproducibility of radiomics features across true pathologies. Third, we did not evaluate interobserver variability in this study. Considering that lesion segmentation affects radiomics features, it is necessary to evaluate reproducibility when real lesions are extracted by multiple observers.

## 5. Conclusions

This study is the first to precisely improve radiomics feature reproducibility by excluding redundant features and experimentally demonstrating reproducibility enhancement with internal and external validations. In conclusion, deep learning-based CT image harmonization can improve the reproducibility of radiomics in abdominal CT and may enhance the development and clinical application of radiomics.

## Figures and Tables

**Table 1:** Characteristics of the datasets.

Dataset	Training and Internal Validation Set (*N* = 117)	External Validation (*N* = 63)
M:F	57:60	38:25
Age (mean ± standard deviation, range)	8.7 ± 5.5 years (2 months–19 years)	12.8 ± 6.8 (2–39 years)
Underlying disease	Abdominal pain (*n* = 15, 12.8%) Tumor follow-up (*n* = 69, 59.0%), others (*n* = 33, 28.2%)	Abdominal pain (*n* = 30, 47.6%) Tumor follow-up (*n* = 69, 33.3%), others (*n* = 12, 19.0%)

Abbreviations: M, male; F, female.

**Table 2:** Detailed CT acquisition parameters of the datasets.

Dataset	Training and Internal Validation (*N* = 142 Exams)	External Validation (*N* = 63 Exams)
Vendor	Siemens	Siemens
Machine	Somatom Force	Somatom Definition Flash
Acquisition type	Helical, dual energy	Helical, dual energy
Tube voltage	70 kVp and Sn150 kVp	80 kV and Sn140 kV
Reference tube current	370 mAs for the 70 kVp tube 93 mAs for the Sn150 kVp tube	270 mAs for the 80 kVp tube 104 mAs for the Sn140 kVp tube
Field of view (mm)	152–355	250–350
Slice thickness	3 mm	3 mm
Pixel	512 × 512	512 × 512
Rotation time (s)	0.25	0.28
Pitch	1.2	1.2
Reconstruction methods	FBP, IR, M40, M60, M80, OPT ^†^	FBP, IR, M40, M70 ^†^
Scan timing	Portal phase	Portal phase

^†^ FBP, filtered back projection; IR, iterative reconstruction; M40/60/80, virtual monoenergetic images at 40/60/80 keV; M40/70, virtual monoenergetic images at 40/70 keV.

**Table 3:** Feature harmonization results in internal and external validations.

***Internal Validation***						
**Region of Interest**	**Liver Parenchyma**	**Spleen**	**Vessel**	**Kidney**	**Muscle**	**Air**
Original	92 (24%)	80 (21%)	139 (36%)	84 (22%)	163 (42%)	365 (94%)
Synthetic	222 (57%)	233 (60%)	298 (77%)	245 (63%)	264 (68%)	343 (89%)
Increase (%)	33%	39%	41%	41%	26%	−5%
***External Validation***						
Original	75 (19%)	68 (18%)	55 (14%)	59 (15%)	92 (24%)	369 (95%)
Synthetic	199 (51%)	239 (62%)	266 (69%)	244 (63%)	212 (55%)	365 (94%)
Increase (%)	32%	44%	55%	48%	31%	−1%

## Data Availability

The datasets generated or analyzed during the study are available from the corresponding author on reasonable request.

## Supplementary Materials

The following supporting information can be downloaded at: https://www.mdpi.com/article/10.3390/bioengineering11121212/s1, Figure S1: Architecture of the generator; Figure S2: Architecture of discriminator network. Ref. [36] is cited in Supplementary Materials.
