# Supplementary material for: Enhancing Radiomics Reproducibility: Deep Learning-Based Harmonization of Abdominal Computed Tomography (CT) Images
Source: Bioengineering (Basel). 2024 Nov 30;11(12):1212. doi: 10.3390/bioengineering11121212 (PMC11673047; doi:10.3390/bioengineering11121212)
Supplement: Supplementary file 1 [file bioengineering-11-01212-s001.zip › bioengineering-3291125-supplementary.pdf]

Supplementary Materials:

Seul Bi Lee [1,†], Youngtaek Hong [2,†], Yeon Jin Cho [1,3,*], Dawun Jeong [2,4], Jina Lee [2,4], Jae Won Choi [1], Jae-Yeon Hwang [1], Seunghyun Lee [1,3], Young Hun Choi [1,3] and Jung-Eun Cheon [1,3,5]

Deep learning architecture and implementation details

In this study, the source images are comprised of six different reconstructed images: filtered back projection (FBP), iterative reconstruction (IR) with a strength of 3, and virtual mono-energetic images with 40 keV (M40), 60 keV (M60), 80 keV (M80), and optimum contrast (OPT) from the dual energy scan. The target is IR with a strength of 3. We randomly sampled a 256 x 256 local patch from the same location in all the reconstructed images. Min-max normalization was applied to rescale the CT values to [0, 1].

The generator (G) is a fully convolutional network consisting of two-dimensional (2D) convolutional layers. The encoded features allow G to learn high-level features from a given image x using two hierarchical feature synthesis (HFS) modules. The HFS was enhanced by replacing the parallel application of the HFS channel and spatial attention modules with an advanced sequential application channel and spatial attention modules [17]. The overall architecture of G is illustrated in supplementary figure 1.

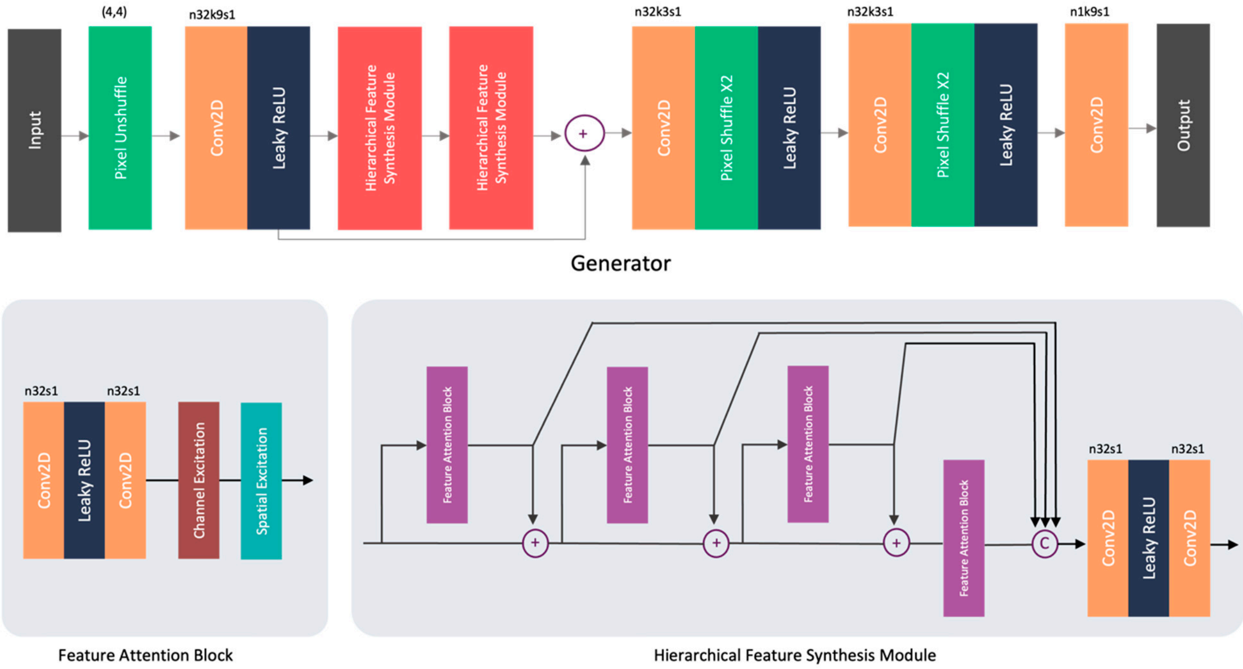

Figure S1. Architecture of the generator with hierarchical feature synthesis module and feature attention block. *k* stands for the size of the convolutional kernel, *n* stands for the number of the kernel, and *s* stands for the size of the convolutional stride.

A local patch was used as an input to the generator network, and the generated image had the same size. We employed a discriminator (D) with a U-Net-like architecture [14, 36]. The discriminator network provides pixel-wise feedback over the local patch. The overall architecture of D is illustrated in supplementary figure 2.

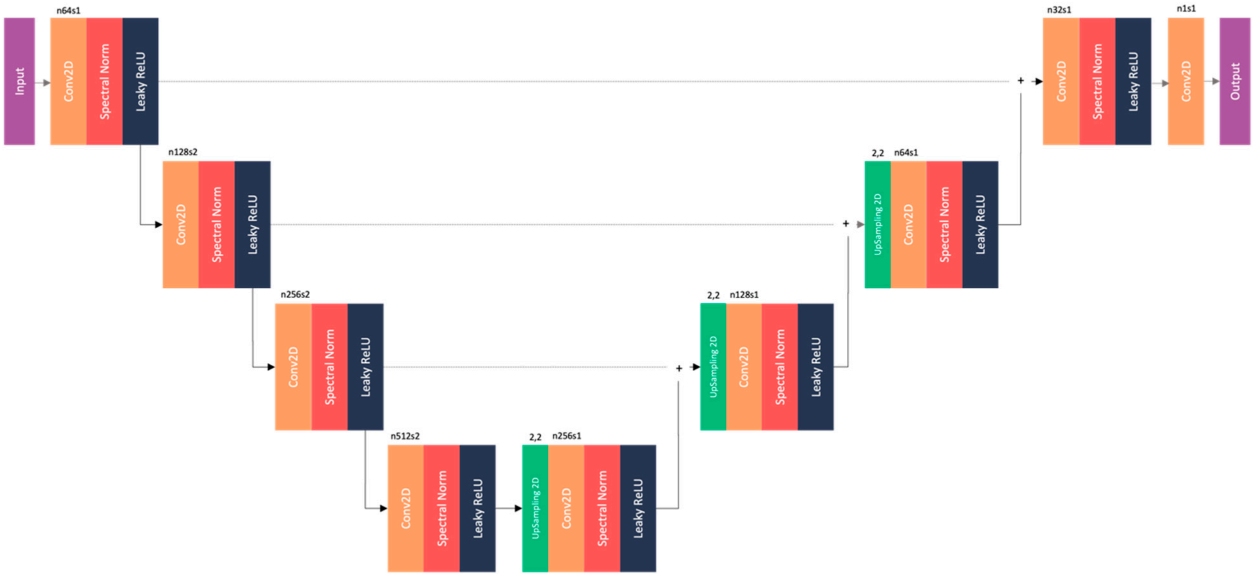

Figure S2. The architecture of discriminator network with corresponding kernel size (k), number of feature maps (n), and stride (s) indicated for the convolutional layer.

The loss function was a combination of three loss functions: 1) L1 loss, 2) perceptual loss, and 3) adversarial loss. The total loss function with a combination coefficient among the loss components is below:

$$L_{total} = (10 \times L_1) + (1 \times L_{perceptual}) + (1 \times L_{adversarial})$$

All parameters of the generator and discriminator networks were optimized using an adaptive moment estimation. The networks were trained for 200 epochs, and the learning rate decayed by 95% of the decay rate after 100 epochs. We implemented this deep learning network using PyTorch version 1.10, (Meta Platforms, California CA, USA). All experiments were performed on a personal computer (Intel i7 9770 (Intel, California CA, USA] with 32 GB of memory) and accelerated using an NVIDIA RTX 2080 Ti GPU (NVIDIA, California CA, USA) with 11 GB of memory.
